# C-Reactive Protein Controls IL-23 Production by Human Monocytes

**DOI:** 10.3390/ijms222111638

**Published:** 2021-10-28

**Authors:** Chiara E. Geyer, Melissa Newling, Lathees Sritharan, Guillermo R. Griffith, Hung-Jen Chen, Dominique L. P. Baeten, Jeroen den Dunnen

**Affiliations:** 1 Center for Experimental and Molecular Medicine, Amsterdam Infection & Immunity Institute, Amsterdam UMC, University of Amsterdam, 1105 AZ Amsterdam, The Netherlands; c.e.geyer@amsterdamumc.nl; 2 Department of Rheumatology & Clinical Immunology, Amsterdam Rheumatology & Immunology Center (ARC), Amsterdam UMC, University of Amsterdam, 1105 AZ Amsterdam, The Netherlands; melissanewling@hotmail.com (M.N.); Lathees11@hotmail.com (L.S.); d.l.baeten@amsterdamumc.nl (D.L.P.B.); 3 Department of Experimental Immunology, Amsterdam Infection & Immunity Institute, Amsterdam UMC, University of Amsterdam, 1105 AZ Amsterdam, The Netherlands; 4 Department of Medical Biochemistry, Experimental Vascular Biology, Amsterdam Cardiovascular Sciences, Amsterdam Infection & Immunity Institute, Amsterdam UMC, University of Amsterdam, 1105 AZ Amsterdam, The Netherlands; g.r.griffith@amsterdamumc.nl (G.R.G.); h.j.chen@amsterdamumc.nl (H.-J.C.)

**Keywords:** C-reactive protein, IL-23, inflammation, monocyte, Fc receptor, Sepsis, inflammatory bowel disease

## Abstract

C-reactive protein (CRP) is an acute-phase protein in humans that is produced in high quantities by the liver upon infection and under inflammatory conditions. Although CRP is commonly used as a marker of inflammation, CRP can also directly contribute to inflammation by eliciting pro-inflammatory cytokine production by immune cells. Since CRP is highly elevated in serum under inflammatory conditions, we have studied the CRP-induced cytokine profile of human monocytes, one of the main innate immune cell populations in blood. We identified that CRP is relatively unique in its capacity to induce production of the pro-inflammatory cytokine IL-23, which was in stark contrast to a wide panel of pattern recognition receptor (PRR) ligands. We show that CRP-induced IL-23 production was mediated at the level of gene transcription, since CRP particularly promoted gene transcription of *IL23A* (encoding IL-23p19) instead of *IL12A* (encoding IL-12p35), while PRR ligands induce the opposite response. Interestingly, when CRP stimulation was combined with PRR ligand stimulation, as for example, occurs in the context of sepsis, IL-23 production by monocytes was strongly reduced. Combined, these data identify CRP as a unique individual ligand to induce IL-23 production by monocytes, which may contribute to shaping systemic immune responses under inflammatory conditions.

## 1. Introduction

C-reactive protein (CRP) belongs to the family of pentraxins and is an acute phase protein in humans, meaning that it is rapidly up-regulated in serum under inflammatory conditions. CRP is predominantly produced by the liver in response to IL-6, following injury, infection or trauma [1]. Baseline serum concentrations of CRP are less than 3 mg/L, but this level may increase to 500 mg/L within 24–48 h from the onset of infection or inflammation [1,2]. Although CRP is used in the clinic as a sensitive but nonspecific marker of inflammation and cardiovascular events, the physiological function of CRP is still not completely understood [3].

There is growing evidence that CRP plays an active role in inflammatory processes and host defense responses. CRP can bind to various pathogens, mainly bacteria, and to damaged or dying cells to promote their phagocytosis by human leukocytes. Several studies have shown that CRP can provide protection against multiple bacteria, including *Streptococcus pneumoniae* [4,5,6,7], *Salmonella enterica serovar Typhimurium* [8], and *Haemophilus influenzae* [9]. CRP can promote phagocytosis by binding to C1q, which activates the classical human complement pathway, or by direct interaction with phagocytic cells by binding to Fc receptors (FcRs) [10,11,12,13,14]. In addition, CRP is involved in the induction of apoptosis and nitric oxide release [15,16,17].

Moreover, CRP is able to induce the production of cytokines. Cytokine induction by myeloid cells is most commonly known to be induced by pattern recognition receptors (PRRs). PRRs recognize conserved foreign structures on pathogens known as pathogen-associated molecular patterns (PAMPs), but can also recognize endogenous structures that are released upon tissue damage or cell death, known as damage-associated molecular patterns (DAMPs) [18]. Recently, it has become clear that CRP can also induce pro-inflammatory cytokines. Although earlier studies are currently considered to be unreliable because of endotoxin contamination [19,20], we and others have shown that CRP stimulation can increase TNF, IL-1β and IL-6 production by human macrophages, PBMCs, and monocytes [21,22,23,24,25].

Recently, a lot of research has been focused on the pro-inflammatory cytokine IL-23. IL-23 is a heterodimeric cytokine consisting of the subunits IL-23p19 and IL-12p40, which are connected via a disulfide bond. The main biological function of IL-23 is considered to be stimulation of antigen presentation by dendritic cells (DCs), T cell differentiation towards T helper 17 (Th17) cells and production of IFNγ [26]. IL-23 also plays an important role in various (chronic) inflammatory disorders, such as inflammatory bowel disease (IBD), psoriasis, spondyloarthritis, and sepsis [27,28,29,30,31,32,33,34,35,36,37]. IL-23 is primarily known to be secreted by monocyte-derived DCs and macrophages. In contrast, IL-23 expression by monocytes is highly restricted, since individual PRR ligands can only induce small amounts of IL-23, and only particular bacterial species, such as *Burkholderia pseudomallei* and *Francisella tularensis*, can elicit IL-23 by monocytes [38,39].

In this study, we identified that CRP, in contrast to other PRR ligands, induces relatively high levels of IL-23 by human monocytes. We show that this IL-23 production is mediated at the level of gene transcription by preferential transcription of *IL23A* instead of *IL12A*, in contrast to most PRR stimuli. Interestingly, while individual stimulation with CRP induces IL-23 production, we also found that CRP-induced IL-23 production by human monocytes is strongly suppressed upon co-stimulation with PRR ligands.

## 2. Results

### 2.1. CRP Induces IL-23 Production by Human Monocytes

**Figure 1:**
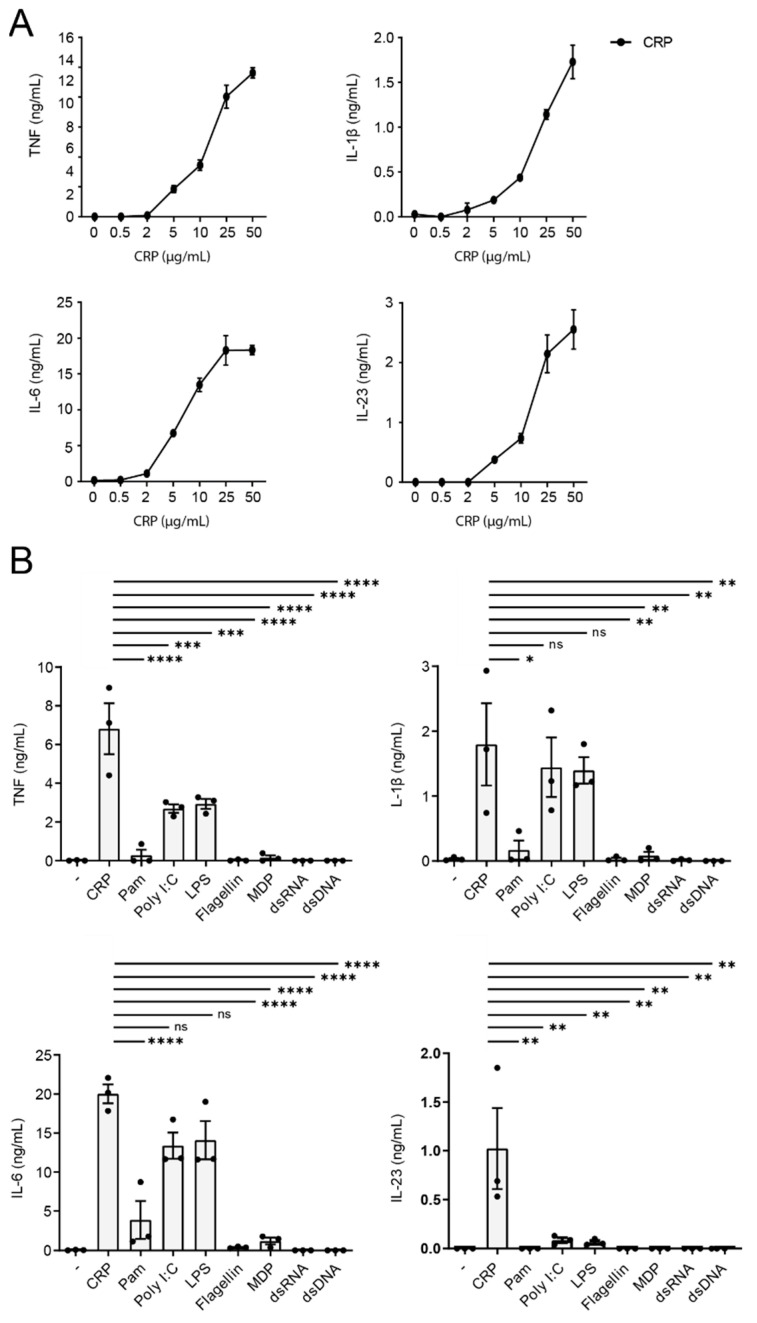
CRP induces IL-23 production by human monocytes. (**A**) Human monocytes were either unstimulated or stimulated with different concentrations of CRP. Supernatants were analyzed after 24 h using ELISA. Representative example from one donor (mean + SEM of triplicate) of three experiments using three different donors. (**B**) Human monocytes were stimulated with CRP, TLR2 ligand Pam3CSK4, TLR3 ligand Poly I:C, TLR4 ligand LPS, TLR5 ligand flagellin, NOD2 ligand MDP, RLR ligand Poly I:C-HMW-LyoVec or CDS ligand Poly(dG:dC)/Lyovec. After 24 h, supernatants were analyzed by ELISA. Pooled data of three different donors. Each dot represents one donor. * *p* < 0.05, ** *p* < 0.01, *** *p* < 0.001, **** *p* < 0.0001, ordinary one-way ANOVA, followed by Tukey’s multiple comparison test.

Since CRP is highly elevated in serum under inflammatory conditions, we have set out to investigate the cytokine profile that is induced by human monocytes, one of the main innate immune cell populations in blood. Therefore, we stimulated human monocytes with a physiologically relevant range of CRP concentrations, from 0.5 µg/mL to 50 µg/mL. As shown in Figure 1A, stimulation with 2 µg/mL or lower levels of CRP induced minimal cytokine production. However, 5 µg/mL or higher levels of CRP increased pro-inflammatory cytokine production in a dose-dependent manner (Figure 1A). Strikingly, not only were previously described cytokines such as TNF, IL-1β, and IL-6 induced, but also IL-23, which is considered to be restrictively expressed by human macrophages and DCs. Next, we determined endotoxin levels of CRP to ascertain that cytokine induction by CRP was not induced by contamination. Endotoxin levels were <0.3 ng/mL, which we verified does not elicit cytokine production (data not shown).

Since PRR ligands normally induce relatively low levels of IL-23 by human monocytes [40], we wanted to compare CRP-induced IL-23 levels with IL-23 levels produced after stimulation with other PRR ligands. To test this, we stimulated monocytes with a variety of PRR ligands, including TLR2 ligand Pam3CSK4 (Pam), TLR3 ligand Poly I:C, TLR4 ligand LPS, TLR5 ligand flagellin, NOD2 ligand MDP, RLR ligand Poly I:C-HMW-LyoVec and CDS ligand Poly(dG:dC)/Lyovec. As a frame of reference, we also assessed TNF, IL-1β, and IL-6 production. Monocytes mainly produced TNF, IL-1β, and IL-6 production in response to CRP, Poly I:C, LPS, and a little to Pam (Figure 1B). However, strikingly, only CRP was able to induce high levels of IL-23 (Figure 1B). All the other PRR ligands were not able to induce IL-23 or induced low levels of IL-23 (Figure 1B).

Together, these data identify CRP as a ligand that induces relatively high levels of IL-23 by human monocytes, suggesting that CRP may contribute to increased IL-23 serum levels under inflammatory conditions (when CRP is elevated).

### 2.2. CRP Favors Gene Transcription of IL23A over IL12A

**Figure 2:**
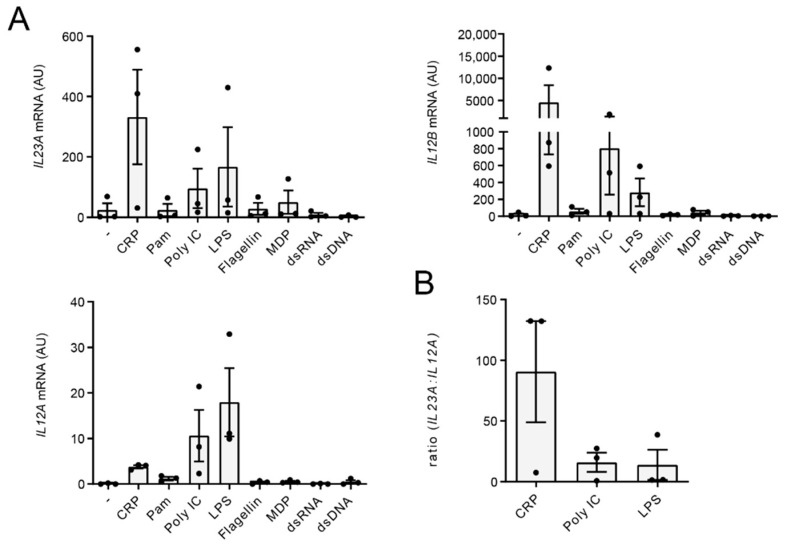
IL-23 production is transcriptionally regulated. Human monocytes were either unstimulated or stimulated with CRP, TLR2 ligand Pam3CSK4, TLR3 ligand Poly I:C, TLR4 ligand LPS, TLR5 ligand flagellin, NOD2 ligand MDP, RLR ligand Poly I:C-HMW-LyoVec or CDS ligand Poly(dG:dC)/Lyovec. At 6 h, mRNA expression was determined by quantitative RT-PCR. (**A**) Data are presented as fold change compared to t = 0 h. (**B**) Ratio *IL23A*:*IL12A* mRNA levels. Pooled data of three different donors. Each dot represents one donor.

Regulation of cytokine production can be orchestrated at different levels. To determine whether the IL-23 production after CRP stimulation is regulated at the level of transcription, we stimulated monocytes with CRP and a variety of other PRR ligands and compared mRNA expression of the two IL-23 subunits, IL-23p19 (encoded by the gene *IL23A*) and IL-12p40 (encoded by the gene *IL12B*). In addition, we assessed the expression of IL-12p35 (encoded by the gene *IL12A*), which pairs with IL-12p40 to form functional IL-12 [41]. Interestingly, when comparing CRP and PRR ligands, we identified that CRP induced relatively higher levels of *IL23A*, while PRR ligands Poly I:C and LPS induced relatively higher levels of *IL12A* (Figure 2A). Since *IL12B* is generally considered to be produced in abundance [42], which is supported by our data that show strong *IL12B* transcription by both CRP and Poly I:C/LPS (Figure 2A), IL-23 and IL-12 production is mostly dependent on transcription of *IL23A* and *IL12A* respectively. Indeed, CRP stimulation induced a higher ration of *IL23A*:*IL12A* mRNA levels than TLR ligands Poly I:C and LPS (Figure 2B). Hence, these data indicate that CRP-induced IL-23 production is regulated at the level of (*IL23A*) gene transcription.

Together, CRP induces IL-23 production by human monocytes through relatively high *IL23A* and low *IL12A* gene transcription compared to PRR stimuli.

### 2.3. CRP-Induced IL-23 Production Is Inhibited upon TLR Co-Stimulation

**Figure 3:**
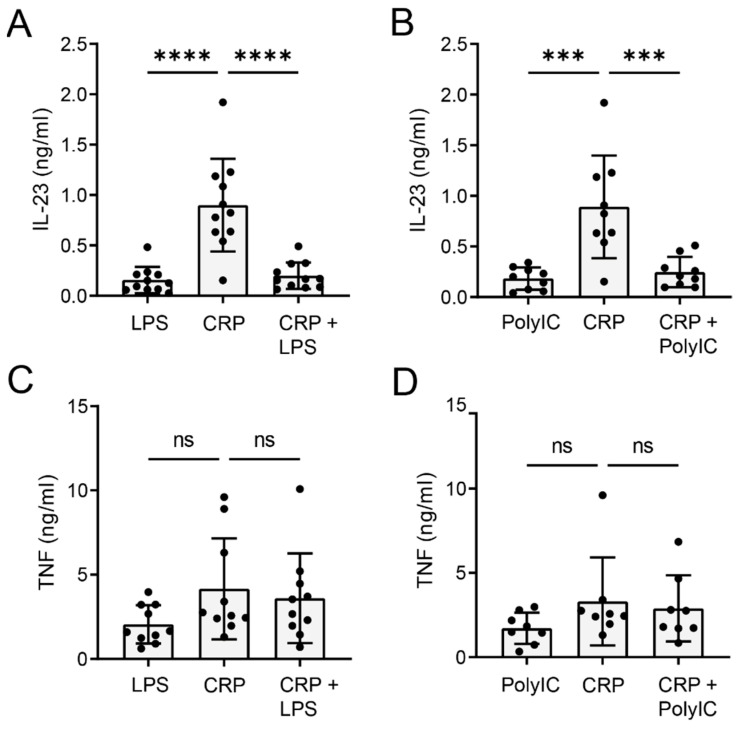
TLR co-stimulation inhibits CRP-induced IL-23 production. Human monocytes were stimulated with CRP, either or not combined with TLR4 ligand LPS (**A**,**C**) or TLR3 ligand Poly I:C (**B**,**D**). After 24 h, IL-23 production was determined by ELISA; pooled data several donors. Each dot represents one donor. *** *p* < 0.001, **** *p* < 0.0001, ordinary one-way ANOVA, followed by Tukey’s multiple comparison test.

Monocytes are primarily located in blood, where they are exposed to elevated CRP levels under inflammatory conditions. However, under particular circumstances, such as systemic infection, monocytes will simultaneously be exposed to microbial stimuli, which activate PRRs such as TLRs. To determine whether TLR activation affects CRP-induced IL-23 production, we simultaneously stimulated human monocytes with CRP and TLR3 ligand Poly I:C or TLR4 ligand LPS. Surprisingly, co-stimulation with Poly I:C or LPS strongly reduced CRP-induced IL-23 production (Figure 3A,B). In contrast, TNF production was not affected by co-stimulation with Poly I:C or LPS (Figure 3C,D, respectively).

Combined, these data show that TLR co-stimulation specifically inhibits CRP-induced IL-23 production by human monocytes.

### 2.4. IL-23 Induction by CRP Is Only Partially Dependent on Syk Signaling

**Figure 4:**
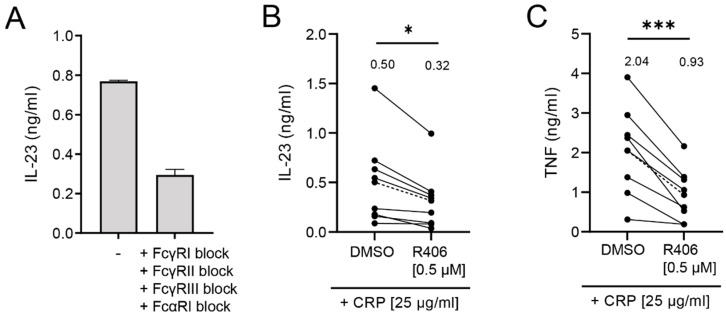
CRP-induced IL-23 production is only partially dependent on Syk signaling. Human monocytes were stimulated with 25 µg/mL CRP. FcR signaling was blocked prior to stimulation by treatment with a mixture of specific antibodies against FcγRI, FcγRII, FcγRIII, and FcαRI (**A**) or by addition of Syk kinase inhibitor R406 [0.5 µM] (**B**,**C**). After 24 h, IL-23 and TNF production was determined by ELISA. Representative example of one donor of four experiments using four different donors (**A**) or pooled data of eight (**B**,**C**) donors. Every pair of dots represents the cytokine induction by one donor, average value indicated by dotted line. * *p* < 0.05, *** *p* < 0.001, paired *t*-test.

To further understand the underlying mechanism by which CRP induces IL-23 production by human monocytes, we investigated the role of FcRs in this process. CRP can interact with multiple FcRs, including Fc gamma receptor (FcγR)I, II, and III, as well as Fc alpha Receptor I (FcαRI) [13,43,44,45], which are all expressed by monocytes [46,47]. To determine if CRP-induced IL-23 production is FcR dependent, we blocked FcR activation using a cocktail of blocking antibodies against FcγRI, II, III, and FcαRI. As shown in Figure 4A, FcR block strongly inhibited CRP-induced production of IL-23 (62% block; from 0.77 to 0.29 ng/mL IL-23), indicating that IL-23 induction by human monocytes is indeed mediated via FcR activation.

One of the key molecules in the signal transduction pathway of both FcγRs and FcαRI is the kinase Syk. In order to identify the role of Syk signaling in CRP-induced IL-23 secretion by human monocytes, we inhibited Syk signaling using the specific Syk inhibitor R406, which we previously showed fully blocks CRP-induced cytokine production by macrophages [20] (Figure 4B,C). Yet, surprisingly, Syk inhibition only partially (36% block; from 0.50 to 0.32 ng/mL IL-23) blocked CRP-induced IL-23 production by monocytes (Figure 4B). In contrast, Syk inhibition more strongly (54% block; from 2.04 to 0.93 ng/mL TNF) suppressed CRP-induced TNF production (Figure 4C).

These findings indicate that CRP-induced IL-23 production by human monocytes is mainly dependent on FcR activation, but only partially dependent on Syk signaling.

## 3. Discussion

Monocytes are generally considered to be restricted in their capacity to produce the pro-inflammatory cytokine IL-23. Yet, in this study we identified that human monocytes produce high levels of IL-23 upon exposure to physiologically relevant concentrations of CRP. We show that this is regulated at the level of gene transcription, since CRP induces the transcription of both subunits of IL-23 (i.e., IL-23p19 and IL-12p40), in contrast to PRR stimuli. Intriguingly, co-stimulation with CRP and PRR ligands strongly suppresses IL-23 production, indicating intricate control of IL-23 production by human monocytes.

CRP is in widespread clinical use as a general marker of inflammation. However, there is growing evidence that CRP may not just be a marker of inflammation, but also plays an active role in inflammatory processes, which includes the induction of pro-inflammatory cytokines [11]. Although it has previously been shown that CRP can induce TNF, IL-1β, and IL-6 by human monocytes [22,25], its capacity to induce IL-23 was still unknown. Here, our data indicate that under inflammatory conditions, during which CRP levels in blood are elevated, human monocytes are activated to produce high levels of IL-23.

Since IL-23 production by human monocytes is generally restricted, a relevant question is what the physiological function of CRP-induced IL-23 production would be. The most well-known function of IL-23 is that it promotes Th17 differentiation and proliferation. Th17 cells play an important role in host defense responses by shaping immunity against extracellular pathogens, such as bacteria and fungi [48]. Since CRP serum levels are particularly elevated during bacterial infections, CRP-induced IL-23 production may provide a rapid mechanism to shape systemic anti-bacterial/fungal immunity upon infection.

Although the abovementioned hypothesis may hold true for the majority of local bacterial infections, the situation is probably different when bacteria enter the bloodstream, e.g., as occurs in cases of sepsis. Interestingly, our data demonstrate that co-stimulation of monocytes with microbial structures such as LPS, which also occurs when bacteria enter the bloodstream, strongly suppresses CRP-induced IL-23 production. The reason for this IL-23 suppression after CRP and LPS co-stimulation remains speculative, but it could be a mechanism to prevent over-activation of the immune system. Bacterial invasion of the bloodstream is a major risk for excessive immune activation as observed during sepsis, and under these conditions the downregulation of IL-23 may help to attenuate pathological inflammation. Indeed, high IL-23 levels are considered to be detrimental during sepsis, and inhibition of the IL-23/Th17 axis may serve to attenuate complications [35,37,49,50]. The molecular mechanism that is responsible for suppression of CRP-induced IL-23 production by LPS in monocytes is yet unclear. Although still speculative, this may be related to the transcription factors that are activated by TLR signaling. These transcription factors may, for example, bind with higher affinity to the *IL23A* promoter (thereby outcompeting the transcription factors that are activated upon stimulation with CRP), while having a reduced capacity to induce transcription initiation (thereby decreasing *IL23A* gene transcription). Another potential explanation could be that TLR signaling induces posttranslational modifications (e.g., phosphorylation, acetylation, or ubiquitination) of the CRP-induced transcription factors, which reduces their *IL23A* translational capacity.

Remarkably, while CRP-LPS co-stimulation strongly suppresses IL-23 by monocytes, we previously identified that CRP-LPS co-stimulation has the exact opposite effect on human macrophages, where co-stimulation strongly promotes the production of pro-inflammatory cytokines, including IL-23 [21]. This difference may be related to the location of the different immune cells. While monocytes in blood control systemic immune responses, macrophages mainly control immune responses in local tissues. In contrast to monocytes that need to prevent systemic over-activation, immune activation by macrophages is restricted to local tissues, and therefore less dangerous to the body as a whole. Another difference between monocytes and macrophages are the specific conditions that are required to induce cytokine production by CRP. While monocytes respond to soluble CRP, macrophages only respond to CRP that is bound to its ligand and thereby forms CRP complexes [21]. Therefore, although upon infection (soluble), CRP levels are elevated almost everywhere in the body, CRP will only activate macrophages in tissues where CRP is bound to its ligands, such as particular strains of bacteria.

In addition to its physiological function, CRP-induced IL-23 production could also play a role in the induction of pathological immune responses. IL-23 is strongly implicated in the pathogenesis of several chronic inflammatory disorders, including IBD, spondyloarthritis, and psoriasis [29,30,32,33,34,51,52]. All of these diseases are also characterized by chronically elevated CRP levels in serum [53,54,55,56,57]. Since microbial stimuli are generally not present in circulation of these patients, these CRP levels may therefore further amplify IL-23 production through continuous activation of monocytes.

Mechanistically, CRP-induced IL-23 production by monocytes is most likely regulated at the level of gene transcription. Both CRP and PRR ligands induce IL-12p40. However, CRP induces relatively high amounts of IL-23p19 (which pairs with IL-12p40 to form functional IL-23), while PRR ligands induce relatively high amounts of IL-12p35 (which pairs with IL-12p40 to form functional IL-12). This shift in IL-12/IL23 balance is quite unique for CRP, since almost all known individual stimuli are poor IL-23 inducers [40]. Since the effect of CRP is regulated at the level of gene transcription, CRP is most likely able to activate a (combination of) transcription factor(s) that PRRs do not, and which can efficiently transcribe the *IL23A* gene. Currently, very little is known about which transcription factors are activated by CRP. However, c-Rel is known to be important for *IL23A* transcription and therefore could be involved [58]. In addition, IRF5 is a positive regulator of *IL23A* transcription [59]. Interestingly, we recently identified that stimulation of FcγRIIa by IgG immune complexes activates IRF5 [60]. Since we have shown that FcγRIIa is one of the main cytokine-inducing receptors of CRP on human macrophages [21], IRF5 may also be induced in monocytes to increase IL-23 production.

Since CRP-induced IL-23 production by monocytes was dependent on FcR activation, it was surprising that IL-23 induction was mostly independent of Syk signaling. Nearly all previously described processes induced by CRP-binding FcRs (i.e., FcγRI, IIa, III, and FcαRI) are dependent on Syk [61,62]. Recently also a Syk-independent “non-canonical” pathway has also been described in dendritic cells for FcγRIIa, which is activated in parallel with Syk-dependent signaling [63]. Whether the same pathway is responsible for CRP-induced IL-23 production by monocytes is still unclear, but these findings provide additional evidence for non-canonical FcR signaling in human immune cells. In addition, targeting this yet unknown pathway may provide new options for therapeutic immunomodulation in the future.

Together, these data indicate that CRP controls IL-23 production by human monocytes, and thereby may contribute to shaping immunity during infection, but perhaps also worsen the pathology of IL-23-associated chronic inflammatory disorders.

## 4. Materials and Methods

### 4.1. Cell Stimulation

PBMCs used in this study were derived from buffy coats obtained from healthy donors, as anonymously provided by Sanquin Blood Supply, Amsterdam. PBMCs were isolated from heparinized peripheral blood by density gradient centrifugation on Lymphoprep (Nycomed, Oslo, Norway). Monocytes were isolated from PBMCs by MACS isolation using CD14 microbeads (Miltenyi Biotec, Bergisch Gladbach, Germany) as previously described [64].

Cells were stimulated in 96-well culture plates (40,000–50,000 cells/well) with 25 µg/mL CRP purified from human plasma (>99% purity and tested negative for infectious agents; Sigma–Aldrich, Darmstadt, Germany). Endotoxin levels of CRP were determined by the Limulus Amebocyte Lysate Chromogenic Endpoint Assay (Hycult Biotech, Uden, The Netherlands). Furthermore, cells were stimulated with 10 µg/mL Pam3CSK4 (Pam; InvivoGen, Toulouse, France), 20 µg/mL Poly I:C (Sigma–Aldrich, Darmstadt, Germany), 100 ng/mL LPS (Escherichia coli o111:B4; Sigma Aldrich, Darmstadt, Germany), 1 µg/mL flagellin, 10 µg/mL MDP, 1 µg/mL Poly I:C-HMW-Lyovec or 10 µg/mL Poly (dG:dC)/Lyovec (all InvivoGen, Toulouse, France).

For Syk kinase inhibition, cells were pre-incubated with 0.5 µM R406 (Selleckchem, Houston, TX, USA), for 30 min at 37 °C. Fc receptor block was achieved by pre-incubating the cells with a mixture of the following antibodies: anti-FcγRI (CD64; 10.1; BD Biosciences, Franklin Lakes, NJ, USA), anti-FcγRII (CD32a; IV.3; Stemcell Technologies, Vancouver, BC, Canada); anti-FcγRIII (CD16; 3G8; BD Biosciences, Franklin Lakes, NJ, USA), anti-FcαRI (CD89; MIP8a; Abcam, Cambridge, UK ) in a concentration of 20 µg/mL for 30 min at 4 °C. After the pre-incubation, the solution was diluted with culture media to receive a final antibody concentration of 5 µg/mL.

### 4.2. ELISA

For analysis of cytokine production, supernatants were harvested after overnight stimulation and stored at −20 °C. Cytokine levels in supernatants were measured by enzyme-linked immunosorbent assay (ELISA), using the following antibody pairs: TNF (MAb1; MAb11; eBioscience, San Diego, CA, USA), IL-1β (CT213-c; CT213-d), IL-6 (CT205-c; CT205-d), and IL-23 (CT517-c; CD517-d; all U-CyTech, Utrecht, The Netherlands).

### 4.3. Quantitative RT-PCR

For mRNA-level analysis, monocytes were lysed at several time points; t = 0 h; t = 1.5 h; t = 3 h or t = 6 h, after which mRNA was extracted using the RNeasy Mini Kit (Qiagen, Venlo, The Netherlands). cDNA was synthesized using the RevertAid H Minus First Strand cDNA Synthesis Kit (Thermo Fisher Scientific, Waltham, MA, USA). Quantitative RT-PCR was performed on StepOnePlusTM Real-Time PCR System (Applied Biosystems, Thermo Fisher Scientific, Waltham, MA, USA) using Taqman gene expression assays for *IL23A* (Hs00372324_m1), *IL12B* (Hs01011518_m1), *IL12A* (Hs01073447_m1), and *GAPDH* (4310884E) according to the protocol of the manufacturer (Thermo Fisher Scientific, Waltham, MA, USA). mRNA levels were normalized to Ct-values of the housekeeping gene GAPDH, and folds were calculated compared with an unstimulated control sample (t = 0 h).

### 4.4. Data Analysis

Data were analyzed for statistical significance using ordinary one-way ANOVA, followed by Tukey’s multiple comparison test or paired *t*-test as indicated. Analysis was performed using with GraphPad Prism version 7 software (GraphPad Software, San Diego, CA, USA).

## Data Availability

Not applicable.
